# Tetra-μ_3_-selen­ido-1:2:3κ^3^ *Se*;1:2:4κ^3^ *Se*;1:3:4κ^3^ *Se*;2:3:4κ^3^ *Se*-tetrakis­[(η^5^-methyl­cyclo­penta­dien­yl)molybdenum(III)](6 *Mo—Mo*)

**DOI:** 10.1107/S2414314623006570

**Published:** 2023-08-01

**Authors:** Akito Ouchi, Tsugiko Takase, Shinji Inomata

**Affiliations:** a Faculty of Symbiotic Systems Science, Fukushima University, 1 Kanayagawa, Fukushima 960-1296, Japan; University of Aberdeen, United Kingdom

**Keywords:** crystal structure, molybdenum cluster, selenide ligand

## Abstract

The title cluster compound, [Mo_4_(η^5^-C_5_H_4_Me)_4_(μ_3_-Se)_4_], was synthesized from the reaction of [Mo(η^5^-C_5_H_4_Me)(CO)_3_]_2_ with grey selenium in refluxing xylene solution under a nitro­gen atmosphere. The complete cluster is generated by a crystallographic twofold axis and contains an Mo_4_Se_4_ cubane-like core surrounded by four η^5^-methylcyclo­pentadienyl ligands. In the core, the four molybdenum atoms are connected to each other to form a tetra­hedron, with a selenium atom capping each face. The Mo—Mo bond lengths vary from 2.9857 (5) to 3.0083 (3) Å and the Mo—Se separations range from 2.4633 (4) to 2.4693 (5) Å.

## Abstract 2

The title cluster contains an Mo tetra­hedron capped on each face with a μ_3_-selenide ligand.

## Graphical abstract

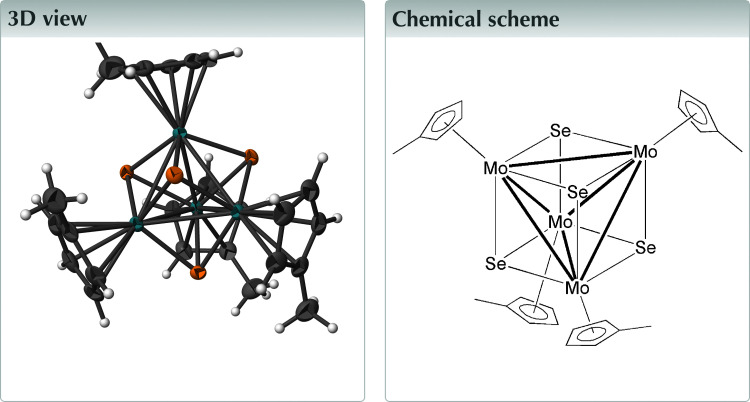

## Structure description

**Figure 1:**
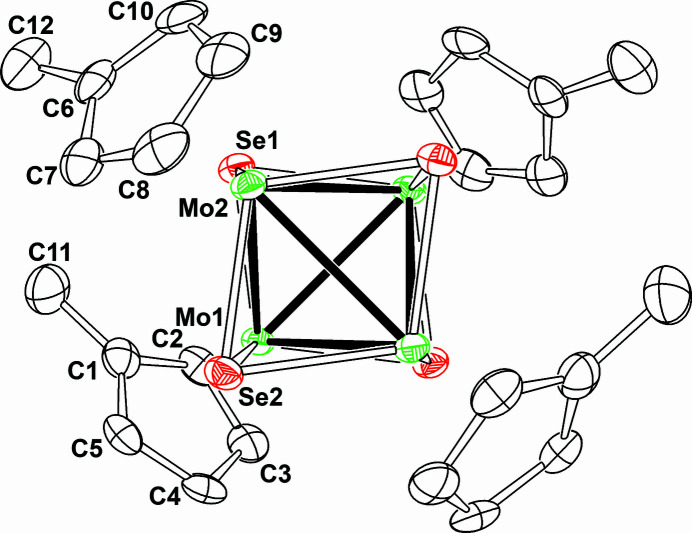
A view of the mol­ecular structure of the title compound, with the asymmetric atoms labeled; unlabelled atoms are generated by the symmetry operation −*x*, *y*, 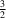 − *z*. Hydrogen atoms are omitted for charity. Displacement ellipsoids are drawn at the 50% probability level.

In comparison to many studies on transition-metal sulfur cubane-type clusters, which contain an *M*_4_S_4_ core, those on selenium analogues are relatively rare. One of the reasons for this rarity could be caused by the insolubility of grey selenium to common organic solvents, as well as water, which are employed for synthesis. We found that grey selenium easily reacts with the organometallic molybdenum compound [Mo(η^5^-C_5_H_4_Me)(CO)_3_]_2_ in organic media to produce a new molybdenum-selenium cubane-type cluster [Mo_4_(η^5^-C_5_H_4_Me)_4_(μ_3_-Se)_4_]. We now report the structural details of the cluster. The partially labeled mol­ecular structure of the title compound is shown in Fig. 1 ▸. The cluster possess twofold symmetry because of the existence of twofold axis through the cluster. The cluster has a distorted-cubane type Mo_4_Se_4_ core surrounded by four methyl­cyclo­penta­dienyl ligands. In the core, four molybdenum atoms are connected by each other through six Mo—Mo bonds to give a molybdenum tetra­hedron. The distances of the Mo—Mo bonds range from 2.9857 (5) to 3.0083 (3) Å, which are somewhat longer than those in [Mo_4_(H_2_O)_12_(μ_3_-Se)_4_](MeC_6_H_4_SO_3_)_5_·15H_2_O [mean value 2.865 (4) Å; Henkel *et al.*, 1990 ▸] and (NH_4_)_6_[Mo_4_(CN)_12_(μ_3_-Se)_4_]·6H_2_O [2.886 (4) Å, *T_d_* symmetry; Virovets *et al.*, 2000 ▸]. However, the Mo—Mo distances in the title compound are quite close to those in an isoelectronic cluster [Mo_4_(η^5^-C_5_H_4_Pr^i^)_4_(μ_3_-Se)_4_] (mean value of 2.9870 Å), which was synthesized by the reaction of [Mo_2_(η^5^-C_5_H_4_Pr^i^)_2_(μ-Cl)_4_] with LiSeH (Baird *et al.*, 1991 ▸). On each face of the Mo tetra­hedron, a selenium atom is located. The Mo—Se distances are in the range 2.4633 (4) to 2.4693 (5) Å and are normal.

## Synthesis and crystallization

A xylene solution (30 ml) of [Mo(η^5^-C_5_H_4_Me)(CO)_3_]_2_ (519 mg, 1.00 mmol) and grey selenium (180 mg, 2.28 mmol) was refluxed for 17 h under a nitro­gen atmosphere. The color of the solution gradually changed from red to brown. After removal of excess selenium by filtration, evaporation of the solvent from the filtrate gave a purple–brown solid. Crystallization was performed by use of the mixed solvents CH_2_Cl_2_/diethyl ether (1:2 *v*/*v*). Yield: 300 mg (59%). A single-crystal suitable for X-ray analysis was selected from the crystallized sample.

## Refinement

Crystal data, date collection and structure refinement details are summarized in Table 1 ▸.

## Supplementary Material

Crystal structure: contains datablock(s) global, I. DOI: 10.1107/S2414314623006570/hb4439sup1.cif

Structure factors: contains datablock(s) I. DOI: 10.1107/S2414314623006570/hb4439Isup2.hkl

CCDC reference: 2285076

Additional supporting information:  crystallographic information; 3D view; checkCIF report

## Figures and Tables

**Table 1:** Experimental details

Crystal data	
Chemical formula	[Mo_4_(C_6_H_7_)_4_Se_4_]
*M*_r_	1016.09
Crystal system, space group	Monoclinic, *C*2/*c*
Temperature (K)	296
*a*, *b*, *c* (Å)	21.1820 (4), 8.42496 (16), 16.4482 (3)
β (°)	120.6613 (7)
*V* (Å^3^)	2524.93 (9)
*Z*	4
Radiation type	Mo *K*α
μ (mm^−1^)	7.72
Crystal size (mm)	0.10 × 0.10 × 0.10

Data collection	
Diffractometer	Rigaku R-AXIS RAPID
Absorption correction	Multi-scan (*ABSCOR*; Rigaku, 1995 ▸)
*T*_min_, *T*_max_	0.201, 0.462
No. of measured, independent and observed [*F* ^2^ > 2.0σ(*F* ^2^)] reflections	11868, 2878, 2617
*R*_int_	0.040
(sin θ/λ)_max_ (Å^−1^)	0.649

Refinement	
*R*[*F* ^2^ > 2σ(*F* ^2^)], *wR*(*F* ^2^), *S*	0.021, 0.051, 1.07
No. of reflections	2878
No. of parameters	147
H-atom treatment	H-atom parameters constrained
Δρ_max_, Δρ_min_ (e Å^−3^)	0.49, −0.79

Computer programs: RAPID AUTO (Rigaku, 2006 ▸), *SIR97* (Altomare *et al.*, 1999 ▸), *SHELXL2018/3* (Sheldrick, 2015 ▸) and *CrystalStructure* (Rigaku, 2010 ▸).

## full crystallographic data

### Crystal data

**Table:**

[Mo_4_(C_6_H_7_)_4_Se_4_]	*F*(000) = 1904.00
*M**_r_* = 1016.09	*D*_x_ = 2.673 Mg m^−^^3^
Monoclinic, *C*2/*c*	Mo *K*α radiation, λ = 0.71075 Å
*a* = 21.1820 (4) Å	Cell parameters from 10275 reflections
*b* = 8.42496 (16) Å	θ = 3.2–27.5°
*c* = 16.4482 (3) Å	µ = 7.72 mm^−^^1^
β = 120.6613 (7)°	*T* = 296 K
*V* = 2524.93 (9) Å^3^	Platelet, brown
*Z* = 4	0.10 × 0.10 × 0.10 mm

### Data collection

**Table:**

Rigaku R-AXIS RAPID diffractometer	2617 reflections with *F*^2^ > 2.0σ(*F*^2^)
Detector resolution: 10.000 pixels mm^-1^	*R*_int_ = 0.040
ω scans	θ_max_ = 27.5°, θ_min_ = 3.3°
Absorption correction: multi-scan (ABSCOR; Rigaku, 1995)	*h* = −24→27
*T*_min_ = 0.201, *T*_max_ = 0.462	*k* = −10→10
11868 measured reflections	*l* = −21→21
2878 independent reflections	

### Refinement

**Table:**

Refinement on *F*^2^	Secondary atom site location: difference Fourier map
*R*[*F*^2^ > 2σ(*F*^2^)] = 0.021	Hydrogen site location: inferred from neighbouring sites
*wR*(*F*^2^) = 0.051	H-atom parameters constrained
*S* = 1.07	*w* = 1/[σ^2^(*F*_o_^2^) + (0.0168*P*)^2^ + 4.2366*P*] where *P* = (*F*_o_^2^ + 2*F*_c_^2^)/3
2878 reflections	(Δ/σ)_max_ = 0.002
147 parameters	Δρ_max_ = 0.49 e Å^−^^3^
0 restraints	Δρ_min_ = −0.79 e Å^−^^3^
Primary atom site location: structure-invariant direct methods	

### Special details

**Table:**

Geometry. All esds (except the esd in the dihedral angle between two l.s. planes) are estimated using the full covariance matrix. The cell esds are taken into account individually in the estimation of esds in distances, angles and torsion angles; correlations between esds in cell parameters are only used when they are defined by crystal symmetry. An approximate (isotropic) treatment of cell esds is used for estimating esds involving l.s. planes.
Refinement. Refinement was performed using all reflections. The weighted R-factor (wR) and goodness of fit (S) are based on F^2^. R-factor (gt) are based on F. The threshold expression of F^2^ > 2.0 sigma(F^2^) is used only for calculating R-factor (gt).All hydrogen atoms were placed at calculated positions (C—H = 0.96–0.98 Å) and refined using a riding model with *U*_iso_(H) = 1.2*U*_eq_(C).

### Fractional atomic coordinates and isotropic or equivalent isotropic displacement parameters (Å^2^)

**Table:**

	*x*	*y*	*z*	*U*_iso_*/*U*_eq_	
Mo1	−0.05709 (2)	0.36774 (3)	0.77761 (2)	0.01956 (7)	
Mo2	0.05898 (2)	0.11729 (3)	0.85272 (2)	0.02062 (7)	
Se1	−0.07420 (2)	0.07974 (3)	0.78522 (2)	0.02368 (8)	
Se2	0.07609 (2)	0.40514 (3)	0.88225 (2)	0.02324 (8)	
C1	−0.16873 (18)	0.4032 (4)	0.7760 (2)	0.0334 (7)	
C2	−0.16199 (18)	0.5254 (4)	0.7228 (2)	0.0317 (7)	
H1	−0.197372	0.548621	0.656343	0.038*	
C3	−0.1002 (2)	0.6197 (4)	0.7834 (2)	0.0344 (7)	
H2	−0.086145	0.719615	0.766422	0.041*	
C4	−0.0688 (2)	0.5553 (4)	0.8753 (2)	0.0350 (7)	
H3	−0.028235	0.601956	0.932877	0.042*	
C5	−0.10922 (19)	0.4220 (4)	0.8715 (2)	0.0332 (7)	
H4	−0.101723	0.359419	0.925903	0.040*	
C6	0.0722 (2)	−0.0631 (4)	0.9693 (2)	0.0380 (8)	
H5	0.032142	−0.108311	0.975354	0.046*	
C7	0.1045 (2)	−0.1336 (4)	0.9208 (2)	0.0362 (8)	
H6	0.090569	−0.235528	0.887498	0.043*	
C8	0.16702 (18)	−0.0398 (4)	0.9400 (2)	0.0335 (7)	
C9	0.1704 (2)	0.0874 (4)	0.9965 (2)	0.0381 (8)	
H7	0.209754	0.166436	1.024598	0.046*	
C10	0.1123 (2)	0.0738 (4)	1.0150 (2)	0.0401 (9)	
H8	0.105439	0.139947	1.058922	0.048*	
C11	−0.2285 (2)	0.2840 (5)	0.7442 (3)	0.0524 (10)	
H9	−0.213781	0.202041	0.791047	0.063*	
H10	−0.238348	0.238212	0.685470	0.063*	
H11	−0.272079	0.334778	0.735713	0.063*	
C12	0.2244 (2)	−0.0779 (5)	0.9148 (3)	0.0509 (10)	
H12	0.250562	0.017026	0.917858	0.061*	
H13	0.201080	−0.120411	0.851841	0.061*	
H14	0.258069	−0.154750	0.958472	0.061*	

### Atomic displacement parameters (Å^2^)

**Table:**

	*U*^11^	*U*^22^	*U*^33^	*U*^12^	*U*^13^	*U*^23^
Mo1	0.02236 (13)	0.01507 (12)	0.01911 (12)	0.00142 (9)	0.00902 (10)	−0.00024 (9)
Mo2	0.02366 (13)	0.01577 (12)	0.01921 (12)	0.00191 (9)	0.00860 (10)	0.00260 (9)
Se1	0.02711 (16)	0.01821 (15)	0.02549 (15)	−0.00290 (11)	0.01325 (12)	0.00020 (11)
Se2	0.02603 (16)	0.01916 (14)	0.01959 (14)	−0.00302 (11)	0.00805 (12)	−0.00285 (11)
C1	0.0301 (17)	0.0359 (18)	0.0375 (17)	0.0050 (14)	0.0196 (14)	−0.0026 (14)
C2	0.0305 (17)	0.0322 (17)	0.0314 (16)	0.0127 (14)	0.0151 (13)	0.0036 (14)
C3	0.042 (2)	0.0175 (14)	0.0447 (19)	0.0097 (14)	0.0232 (16)	0.0006 (14)
C4	0.0388 (19)	0.0307 (17)	0.0322 (16)	0.0051 (14)	0.0157 (14)	−0.0109 (14)
C5	0.0404 (19)	0.0358 (18)	0.0283 (15)	0.0116 (15)	0.0210 (14)	0.0031 (14)
C6	0.044 (2)	0.0360 (19)	0.0345 (17)	0.0073 (15)	0.0200 (15)	0.0200 (15)
C7	0.046 (2)	0.0199 (15)	0.0370 (17)	0.0100 (14)	0.0169 (16)	0.0127 (13)
C8	0.0306 (17)	0.0340 (18)	0.0287 (15)	0.0130 (14)	0.0099 (13)	0.0145 (14)
C9	0.0344 (19)	0.0376 (19)	0.0261 (15)	0.0056 (15)	0.0036 (14)	0.0067 (14)
C10	0.051 (2)	0.041 (2)	0.0208 (14)	0.0139 (17)	0.0122 (15)	0.0095 (14)
C11	0.039 (2)	0.055 (2)	0.068 (3)	−0.0036 (19)	0.031 (2)	−0.006 (2)
C12	0.046 (2)	0.052 (2)	0.052 (2)	0.0155 (19)	0.0230 (19)	0.0129 (19)

### Geometric parameters (Å, º)

**Table:**

Mo1—C3	2.332 (3)	C2—C3	1.416 (5)
Mo1—C2	2.339 (3)	C2—H1	0.9800
Mo1—C4	2.355 (3)	C3—C4	1.413 (5)
Mo1—C5	2.356 (3)	C3—H2	0.9800
Mo1—C1	2.370 (3)	C4—C5	1.394 (5)
Mo1—Se2	2.4633 (4)	C4—H3	0.9800
Mo1—Se1	2.4659 (4)	C5—H4	0.9800
Mo1—Se2^i^	2.4689 (3)	C6—C10	1.402 (5)
Mo1—Mo1^i^	2.9857 (5)	C6—C7	1.419 (5)
Mo1—Mo2	2.9875 (3)	C6—H5	0.9800
Mo1—Mo2^i^	2.9935 (3)	C7—C8	1.432 (5)
Mo2—C10	2.337 (3)	C7—H6	0.9800
Mo2—C6	2.350 (3)	C8—C9	1.396 (5)
Mo2—C9	2.352 (3)	C8—C12	1.506 (5)
Mo2—C7	2.356 (3)	C9—C10	1.414 (5)
Mo2—C8	2.387 (3)	C9—H7	0.9800
Mo2—Se2	2.4635 (4)	C10—H8	0.9800
Mo2—Se1	2.4691 (4)	C11—H9	0.9600
Mo2—Se1^i^	2.4692 (4)	C11—H10	0.9600
Mo2—Mo2^i^	3.0083 (5)	C11—H11	0.9600
C1—C2	1.404 (5)	C12—H12	0.9600
C1—C5	1.435 (5)	C12—H13	0.9600
C1—C11	1.487 (5)	C12—H14	0.9600
			
C3—Mo1—C2	35.30 (12)	C7—Mo2—Mo1^i^	145.65 (9)
C3—Mo1—C4	35.09 (12)	C8—Mo2—Mo1^i^	118.70 (8)
C2—Mo1—C4	58.11 (11)	Se2—Mo2—Mo1^i^	52.718 (9)
C3—Mo1—C5	58.16 (12)	Se1—Mo2—Mo1^i^	99.982 (11)
C2—Mo1—C5	58.09 (11)	Se1^i^—Mo2—Mo1^i^	52.606 (9)
C4—Mo1—C5	34.41 (12)	Mo1—Mo2—Mo1^i^	59.894 (10)
C3—Mo1—C1	58.43 (12)	C10—Mo2—Mo2^i^	157.50 (10)
C2—Mo1—C1	34.69 (12)	C6—Mo2—Mo2^i^	127.01 (9)
C4—Mo1—C1	58.08 (12)	C9—Mo2—Mo2^i^	164.17 (9)
C5—Mo1—C1	35.36 (11)	C7—Mo2—Mo2^i^	115.98 (9)
C3—Mo1—Se2	100.84 (9)	C8—Mo2—Mo2^i^	131.90 (8)
C2—Mo1—Se2	136.14 (9)	Se2—Mo2—Mo2^i^	100.132 (8)
C4—Mo1—Se2	85.36 (9)	Se1—Mo2—Mo2^i^	52.472 (10)
C5—Mo1—Se2	105.65 (9)	Se1^i^—Mo2—Mo2^i^	52.471 (10)
C1—Mo1—Se2	140.55 (8)	Mo1—Mo2—Mo2^i^	59.902 (8)
C3—Mo1—Se1	145.37 (9)	Mo1^i^—Mo2—Mo2^i^	59.705 (8)
C2—Mo1—Se1	116.29 (9)	Mo1—Se1—Mo2	74.510 (12)
C4—Mo1—Se1	123.79 (9)	Mo1—Se1—Mo2^i^	74.684 (11)
C5—Mo1—Se1	91.58 (9)	Mo2—Se1—Mo2^i^	75.060 (13)
C1—Mo1—Se1	87.30 (8)	Mo1—Se2—Mo2	74.656 (12)
Se2—Mo1—Se1	103.651 (13)	Mo1—Se2—Mo1^i^	74.507 (12)
C3—Mo1—Se2^i^	94.16 (9)	Mo2—Se2—Mo1^i^	74.730 (11)
C2—Mo1—Se2^i^	84.87 (8)	C2—C1—C5	106.8 (3)
C4—Mo1—Se2^i^	128.63 (9)	C2—C1—C11	128.0 (3)
C5—Mo1—Se2^i^	142.82 (8)	C5—C1—C11	125.1 (3)
C1—Mo1—Se2^i^	110.62 (8)	C2—C1—Mo1	71.44 (19)
Se2—Mo1—Se2^i^	103.560 (13)	C5—C1—Mo1	71.77 (18)
Se1—Mo1—Se2^i^	103.372 (13)	C11—C1—Mo1	125.8 (2)
C3—Mo1—Mo1^i^	114.04 (9)	C1—C2—C3	108.9 (3)
C2—Mo1—Mo1^i^	129.84 (8)	C1—C2—Mo1	73.87 (18)
C4—Mo1—Mo1^i^	126.24 (9)	C3—C2—Mo1	72.07 (17)
C5—Mo1—Mo1^i^	157.28 (9)	C1—C2—H1	125.4
C1—Mo1—Mo1^i^	162.73 (8)	C3—C2—H1	125.4
Se2—Mo1—Mo1^i^	52.834 (10)	Mo1—C2—H1	125.4
Se1—Mo1—Mo1^i^	100.268 (9)	C4—C3—C2	107.4 (3)
Se2^i^—Mo1—Mo1^i^	52.659 (10)	C4—C3—Mo1	73.37 (18)
C3—Mo1—Mo2	152.01 (9)	C2—C3—Mo1	72.64 (17)
C2—Mo1—Mo2	168.55 (9)	C4—C3—H2	126.0
C4—Mo1—Mo2	122.87 (8)	C2—C3—H2	126.0
C5—Mo1—Mo2	115.34 (8)	Mo1—C3—H2	126.0
C1—Mo1—Mo2	134.39 (8)	C5—C4—C3	108.5 (3)
Se2—Mo1—Mo2	52.676 (10)	C5—C4—Mo1	72.81 (18)
Se1—Mo1—Mo2	52.794 (10)	C3—C4—Mo1	71.54 (17)
Se2^i^—Mo1—Mo2	100.565 (11)	C5—C4—H3	125.6
Mo1^i^—Mo1—Mo2	60.153 (8)	C3—C4—H3	125.6
C3—Mo1—Mo2^i^	143.92 (9)	Mo1—C4—H3	125.6
C2—Mo1—Mo2^i^	117.47 (8)	C4—C5—C1	108.4 (3)
C4—Mo1—Mo2^i^	173.63 (9)	C4—C5—Mo1	72.78 (18)
C5—Mo1—Mo2^i^	140.04 (9)	C1—C5—Mo1	72.87 (17)
C1—Mo1—Mo2^i^	115.58 (8)	C4—C5—H4	125.6
Se2—Mo1—Mo2^i^	100.539 (11)	C1—C5—H4	125.6
Se1—Mo1—Mo2^i^	52.709 (9)	Mo1—C5—H4	125.6
Se2^i^—Mo1—Mo2^i^	52.551 (9)	C10—C6—C7	108.1 (3)
Mo1^i^—Mo1—Mo2^i^	59.954 (8)	C10—C6—Mo2	72.11 (18)
Mo2—Mo1—Mo2^i^	60.394 (10)	C7—C6—Mo2	72.68 (17)
C10—Mo2—C6	34.82 (13)	C10—C6—H5	125.8
C10—Mo2—C9	35.10 (13)	C7—C6—H5	125.8
C6—Mo2—C9	57.92 (13)	Mo2—C6—H5	125.8
C10—Mo2—C7	58.26 (13)	C6—C7—C8	107.6 (3)
C6—Mo2—C7	35.11 (12)	C6—C7—Mo2	72.22 (18)
C9—Mo2—C7	57.88 (13)	C8—C7—Mo2	73.65 (18)
C10—Mo2—C8	57.94 (12)	C6—C7—H6	126.0
C6—Mo2—C8	58.10 (12)	C8—C7—H6	126.0
C9—Mo2—C8	34.26 (12)	Mo2—C7—H6	126.0
C7—Mo2—C8	35.14 (12)	C9—C8—C7	107.3 (3)
C10—Mo2—Se2	89.56 (9)	C9—C8—C12	125.0 (3)
C6—Mo2—Se2	122.11 (9)	C7—C8—C12	127.4 (3)
C9—Mo2—Se2	86.33 (9)	C9—C8—Mo2	71.47 (18)
C7—Mo2—Se2	143.71 (9)	C7—C8—Mo2	71.21 (18)
C8—Mo2—Se2	115.33 (9)	C12—C8—Mo2	127.8 (2)
C10—Mo2—Se1	105.64 (10)	C8—C9—C10	109.1 (3)
C6—Mo2—Se1	85.26 (9)	C8—C9—Mo2	74.27 (18)
C9—Mo2—Se1	140.23 (9)	C10—C9—Mo2	71.88 (18)
C7—Mo2—Se1	101.27 (9)	C8—C9—H7	125.3
C8—Mo2—Se1	136.41 (9)	C10—C9—H7	125.3
Se2—Mo2—Se1	103.549 (13)	Mo2—C9—H7	125.3
C10—Mo2—Se1^i^	144.67 (10)	C6—C10—C9	107.9 (3)
C6—Mo2—Se1^i^	130.55 (9)	C6—C10—Mo2	73.08 (18)
C9—Mo2—Se1^i^	112.09 (9)	C9—C10—Mo2	73.01 (18)
C7—Mo2—Se1^i^	96.22 (9)	C6—C10—H8	125.8
C8—Mo2—Se1^i^	86.98 (8)	C9—C10—H8	125.8
Se2—Mo2—Se1^i^	103.434 (13)	Mo2—C10—H8	125.8
Se1—Mo2—Se1^i^	103.004 (13)	C1—C11—H9	109.5
C10—Mo2—Mo1	113.56 (9)	C1—C11—H10	109.5
C6—Mo2—Mo1	121.66 (9)	H9—C11—H10	109.5
C9—Mo2—Mo1	133.12 (9)	C1—C11—H11	109.5
C7—Mo2—Mo1	151.79 (9)	H9—C11—H11	109.5
C8—Mo2—Mo1	167.07 (9)	H10—C11—H11	109.5
Se2—Mo2—Mo1	52.668 (10)	C8—C12—H12	109.5
Se1—Mo2—Mo1	52.696 (9)	C8—C12—H13	109.5
Se1^i^—Mo2—Mo1	100.141 (11)	H12—C12—H13	109.5
C10—Mo2—Mo1^i^	138.65 (10)	C8—C12—H14	109.5
C6—Mo2—Mo1^i^	173.27 (10)	H12—C12—H14	109.5
C9—Mo2—Mo1^i^	115.84 (9)	H13—C12—H14	109.5
			
C5—C1—C2—C3	−0.5 (4)	C10—C6—C7—C8	1.8 (4)
C11—C1—C2—C3	174.6 (3)	Mo2—C6—C7—C8	65.7 (2)
Mo1—C1—C2—C3	−64.0 (2)	C10—C6—C7—Mo2	−63.8 (2)
C5—C1—C2—Mo1	63.5 (2)	C6—C7—C8—C9	−2.0 (3)
C11—C1—C2—Mo1	−121.4 (4)	Mo2—C7—C8—C9	62.8 (2)
C1—C2—C3—C4	−0.5 (4)	C6—C7—C8—C12	171.5 (3)
Mo1—C2—C3—C4	−65.7 (2)	Mo2—C7—C8—C12	−123.7 (3)
C1—C2—C3—Mo1	65.2 (2)	C6—C7—C8—Mo2	−64.7 (2)
C2—C3—C4—C5	1.3 (4)	C7—C8—C9—C10	1.3 (3)
Mo1—C3—C4—C5	−63.9 (2)	C12—C8—C9—C10	−172.4 (3)
C2—C3—C4—Mo1	65.2 (2)	Mo2—C8—C9—C10	63.9 (2)
C3—C4—C5—C1	−1.6 (4)	C7—C8—C9—Mo2	−62.6 (2)
Mo1—C4—C5—C1	−64.7 (2)	C12—C8—C9—Mo2	123.7 (3)
C3—C4—C5—Mo1	63.0 (2)	C7—C6—C10—C9	−1.0 (4)
C2—C1—C5—C4	1.3 (4)	Mo2—C6—C10—C9	−65.2 (2)
C11—C1—C5—C4	−173.9 (3)	C7—C6—C10—Mo2	64.2 (2)
Mo1—C1—C5—C4	64.6 (2)	C8—C9—C10—C6	−0.2 (4)
C2—C1—C5—Mo1	−63.3 (2)	Mo2—C9—C10—C6	65.3 (2)
C11—C1—C5—Mo1	121.4 (3)	C8—C9—C10—Mo2	−65.5 (2)

Symmetry code: (i) −*x*, *y*, −*z*+3/2.
